# Comparative Thermal Tolerance and Tissue-Specific Responses Patterns to Gradual Heat Stress in Reciprocal Cross Hybrids of *Acipenser baerii* and *A. schrenckii*

**DOI:** 10.3390/ijms27010132

**Published:** 2025-12-22

**Authors:** Wei Wang, Linan Gao, Xiaoyu Yan, Wenjie Liu, Tian Dong, Hailiang Song, Guoqing Ma, Hongxia Hu

**Affiliations:** 1 Fisheries Science Institute, Beijing Academy of Agriculture and Forestry Sciences & Beijing Key Laboratory of Fisheries Biotechnology, Beijing 100068, China; wangwei8848@126.com (W.W.);; 2 Key Laboratory of Sturgeon Genetics and Breeding, Ministry of Agriculture and Rural Affairs, Hangzhou 311799, China; 3 Faculty of Fisheries and Protection of Waters, South Bohemian Research Center of Aquaculture and Biodiversity of Hydrocenoses, University of South Bohemia in Ceske Budejovice, Zatisi 728/11, 389 25 Vodnany, Czech Republic

**Keywords:** hybrid sturgeon, Siberian sturgeon, slow heat stress, *hsp* gene, tissue-specific responses, antioxidant system, thermal adaptation, maternal lineage

## Abstract

The increasing frequency and intensity of heatwaves due to climate change pose significant challenges to sturgeon aquaculture. This study investigated the effects of gradual heat stress (1 °C every 8 h) on two reciprocal hybrid sturgeon strains (*Acipenser baerii* ♀ × *A. schrenckii* ♂, (BS hybrid); *A. schrenckii* ♀ × *A. baerii* ♂, (SB hybrid)), focusing on their antioxidant defense mechanisms, heat shock protein (HSP) expression, and liver and gill tissue histology. When water temperature raised to 34.3 °C (about 104 h), LOE (loss of equilibrium) individuals appeared. Twenty-four hours after sampling, fifteen BS hybrid sturgeon remained alive, whereas no SB hybrid sturgeon survived. In this study, the slow heat stress significantly elevated the expression of HSP-related genes (*hsc70*, *hsp70*, *hsp90*) in both the liver of BS hybrid sturgeon and the gills of SB hybrid sturgeon. However, in the gills of BS hybrid sturgeon and the liver of SB hybrid sturgeon, the expression of *hsp* family genes in the experimental groups was either lower than or comparable to the control group. Significant liver damage, including cellular vacuolization and necrosis, was observed in BS hybrids, while SB hybrid sturgeon exhibited more pronounced gill tissue damage. Among the four antioxidant enzymes—superoxide dismutase (SOD), lactate dehydrogenase (LDH), catalase (CAT) glutathione peroxidase (GPx)—only LDH activity was elevated in the hepatic tissue of BS hybrid sturgeon, corresponding to increased serum lactate levels, while gill LDH activity was higher in SB hybrid sturgeon. In both hybrids, LDH activity exhibited an increasing trend in the kidney. However, total antioxidant capacity (T-AOC) remained unchanged across all three tissues. Both plasma cortisol and lactate were substantially affected by thermal stress. MDA remained at a relatively stable level after heat stress and recovery. These results demonstrate differential tissue-specific responses to heat stress in the reciprocal hybrids. More importantly, the BS hybrid sturgeon exhibited significantly higher thermal tolerance and post-stress survival compared to the SB hybrid sturgeon. These findings reveal that the choice of maternal parent is a critical factor influencing heat resistance in these hybrids, providing a key basis for selective breeding programs and optimizing aquaculture management.

## 1. Introduction

Sturgeons (Acipenseridae), heralded as “living fossils” represent some of the most ancient species within the non-teleost Actinopterygii. These species thrive in cold-temperate waters of the northern hemisphere, with optimal reproductive temperatures generally below 20 °C [1]. However, current climate predictions indicate that heatwaves will become more frequent, prolonged, and intense as global surface temperatures continue to rise, leading to significant increases in aquatic temperature [2]. Such changes pose severe threats to the natural habitats of sturgeons, altering their historical distribution patterns significantly [3].

Recent studies reported that water temperatures in sturgeon farms located in Uruguay, Turkey, and Russia have risen to between 28–30 °C [4,5,6], exceeding the species’ typical thermal tolerance range. These elevated temperatures have triggered numerous physiological stress responses in sturgeons, including decreased feeding rates, slowed growth, and reduced immunity and stress resistance [4,7]. Moreover, such conditions favor the outbreak of pathogens like *Aeromonas hydrophila* [4,8], *A. veronii* [8], *Veronaea botryose* [9], and various sturgeon mimiviruses [10], significantly impacting the health and viability of sturgeon populations.

To assess thermal tolerance in aquatic organisms, the Critical Thermal Maximum (CTMax) is commonly applied as an indicator, defined as the pre-lethal temperature threshold beyond which organisms lose vital functions. [11,12]. However, the ecological relevance of CTMax has been questioned due to the artificially rapid heating rates commonly employed in these assessments, typically around 0.3 °C/min, which often exceed natural rates of temperature increase [13]. Heat tolerance threshold during conventional CTMax tests is generally attributed to cardiac or neurological dysfunction, potentially caused by inadequate oxygen transport [14]. Studies using slower heating rates have frequently reported lower thermal tolerance than those derived from conventional CTMax protocols [15,16].

In response to these challenges, the aquaculture industry is increasingly turning to the development and cultivation of hybrids. Reciprocal hybridization has been widely proven as an effective strategy in aquaculture to exploit heterosis and maternal effects for enhancing environmental stress resistance and disease tolerance [17,18]. For instance, reciprocal crosses in catfish and salmonids have demonstrated significant improvements in hypoxia tolerance and pathogen resistance compared to their parental species [17,18]. In sturgeon hybrids, such as *Acipenser baerii* (Siberian sturgeon) ♀ × *A. schrenckii* (Amur sturgeon) ♂, represented by the new Chinese aquatic variety “Jinglong No. 1”, have shown not only faster growth rates and earlier sexual maturation but also better overall viability compared to their parent species [19,20]. Currently, hybrid sturgeon account for approximately 35% of global sturgeon meat production and 20% of caviar production [21], with over 80% of sturgeon farming in China relying on these robust breeds [22].

The physiological challenges posed by high temperatures, particularly oxidative stress and tissue damage in essential organs like the gills, liver, and kidneys, are well-documented [23]. The gills are vital for gas exchange and ion regulation, while the liver serves as a key organ for metabolic regulation and detoxification, both of which are crucial for managing the oxidative stress induced by high temperatures [4,24]. Similarly, the kidney’s role in waste excretion and osmotic balance is critical, with heat stress potentially disrupting these functions [25]. To understand the molecular mechanisms of thermal adaptation, it is crucial to examine specific stress-response pathways. The Heat Shock Protein (HSP) family acts as the primary cellular defense against thermal damage. HSP70 (inducible) and HSP90 facilitate protein folding and prevent aggregation under stress, while HSC70 (constitutive) maintains housekeeping functions [26,27]. Their transcription is master-regulated by Heat Shock Factor 1 (HSF1) [28]. Physiologically, the stress response involves the neuroendocrine activation of the Hypothalamus–Pituitary–Inter-renal (HPI) axis, leading to the release of cortisol, which mobilizes energy substrates like glucose and lactate to meet increased metabolic demands. Furthermore, oxidative stress induced by heat requires a coordinated antioxidant defense, primarily involving enzymes such as superoxide dismutase (SOD), catalase (CAT), and glutathione peroxidase (GPx), total antioxidant capacity (T-AOC) to neutralize reactive oxygen species (ROS).

Few studies have utilized slower heating rates, which may better reflect natural environmental conditions, to assess the tolerance to extreme temperatures of commercially farmed sturgeon [29]. This study diverges from other research on acute thermal stress in sturgeons [30,31,32], aiming to simulate natural temperature increments at a slow heating rate of 1 °C every 8 h. This approach provided a deeper investigation into the physiological and molecular responses of the *A. baerii* ♀ × *A. schrenckii* ♂ hybrid to elevated temperatures. We assessed the impact of slow heat shock on their antioxidant systems, comparing these hybrids to *A. schrenckii* ♀ × *A. baerii* ♂ counterparts. By evaluating enzyme activities, such as SOD, lactate dehydrogenase (LDH), CAT, GPx, T-AOC, along with MDA (malondialdehyde) levels in liver, gill, and kidney tissues and examining changes in cortisol, glucose, lactate in serum, and heat shock protein-related gene expression, this research will significantly enrich our understanding of the biological resilience of Acipenseridae to thermal stress. These findings will also provide vital reference data for optimizing temperature regulation in the industrial cultivation of hybrid sturgeons, thereby enhancing aquaculture productivity. Furthermore, by comparing the tolerance differences between reciprocal crosses, this study aims to assess the impact of parental lineage (maternal vs. paternal) on thermal resilience, providing a scientific basis for future selective breeding of heat-tolerant sturgeon varieties.

## 2. Results

**Figure 1:**
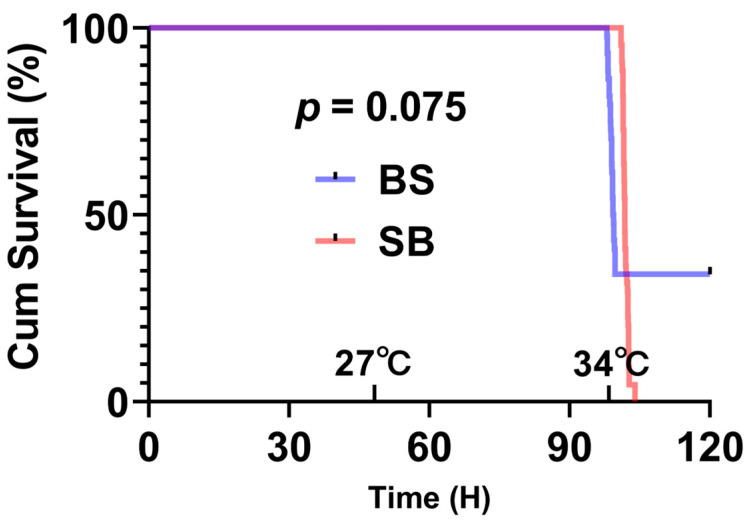
Kaplan–Meier survival curves of BS and SB hybrid sturgeon under slow heat stress. The analysis included n = 44 fish per group (excluding the control and active sampling). The BS hybrid showed a significantly higher survival probability compared to the SB hybrid (*p* < 0.05, log-rank test).

The mean weights were 430.00 ± 39.48 g in control (C), 422.25 ± 52.96 g in LOE group (E1); 410.75 ± 60.27 g in active group (E2) of BS and 423.25 ± 39.81 g in control (C), 383.25 ± 46.78 g in LOE group (E1); 374.43 ± 35.39 g in active group (E2) of SB. There was no significant difference between groups (*p* = 0.747 and *p* = 0.075, respectively). After approximately 104 h, when the water temperature raised to 34.3 °C, the first LOE individual of BS appeared, with the eighth sample reaching LOE at 34.8 °C. In the SB group, the first LOE individual appeared at 34.8 °C, and the eighth sample reached LOE at 35 °C. In addition to collected eight samples of LOE group, 21 BS (35%) and 34 SB (56.7%) individuals in the LOE (Loss of Equilibrium) group experienced sequential mortality. After sampling, the active group comprised 15 BS (25%) and 2 SB (3%) individuals; however, both SB individuals subsequently died within 24 h. The survival curve statistics are significantly different (*p* < 0.05), provided as Figure 1.

### 2.1. Changes in Hsp-Related Gene Expression in the Liver Under Slow Heat Stress

**Figure 2:**
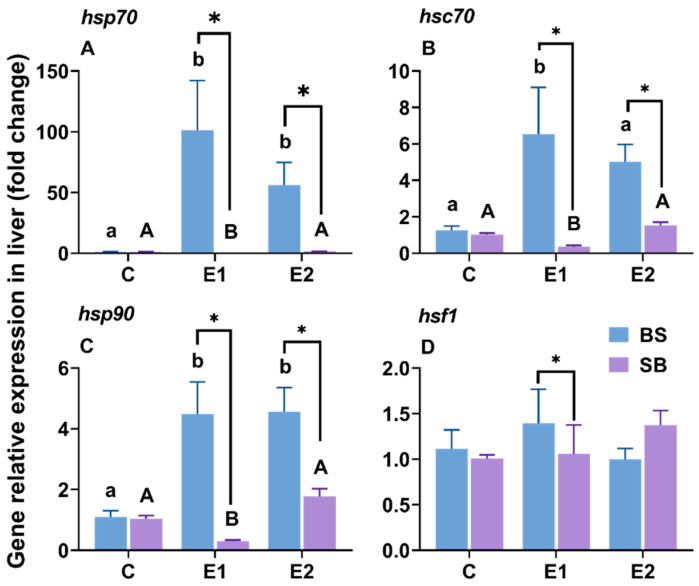
Relative mRNA expression of (**A**) *hsp70*, (**B**) *hsc70*, (**C**) *hsp90*, and (**D**) *hsf1* in liver of BS and SB hybrid sturgeon under slow heat stress. Mean values (±SEM) are shown for every group (n = 8). C: control, E1: LOE group, E2: active group. Significant differences within the BS hybrid sturgeon are denoted by different lowercase letters, while uppercase letters represent significant differences within the SB hybrid sturgeon, *p* < 0.05. Statistically significant differences (*p* < 0.05) between different hybrid sturgeon within the same group are marked by asterisks, as determined by the *t*-test.

In the liver of BS hybrid sturgeon, the expression of HSP family genes (*hsp70*, *hsc70*, and *hsp90*) was significantly upregulated in both heat-stressed groups (E1 and E2) compared to the control (Figure 2A–C). Notably, *hsp70* showed a dramatic 100-fold increase in the LOE group. In contrast, SB hybrids exhibited a significant downregulation or no change in these genes under heat stress. The expression of the transcription factor *hsf1* showed no statistically significant differences among groups in either hybrid (Figure 2D). Both the LOE and active groups showed significantly higher expression of the three *hsp* family genes in BS hybrid sturgeon than in SB hybrid sturgeon (consistent *p*-values, *p* < 0.05) (Figure 2A–C). Only in the LOE group was hsf1 expression elevated in BS hybrid sturgeon compared to SB hybrid sturgeon (Figure 2D).

### 2.2. Changes in Hsp-Related Gene Expression in the Gill Under Slow Heat Stress

**Figure 3:**
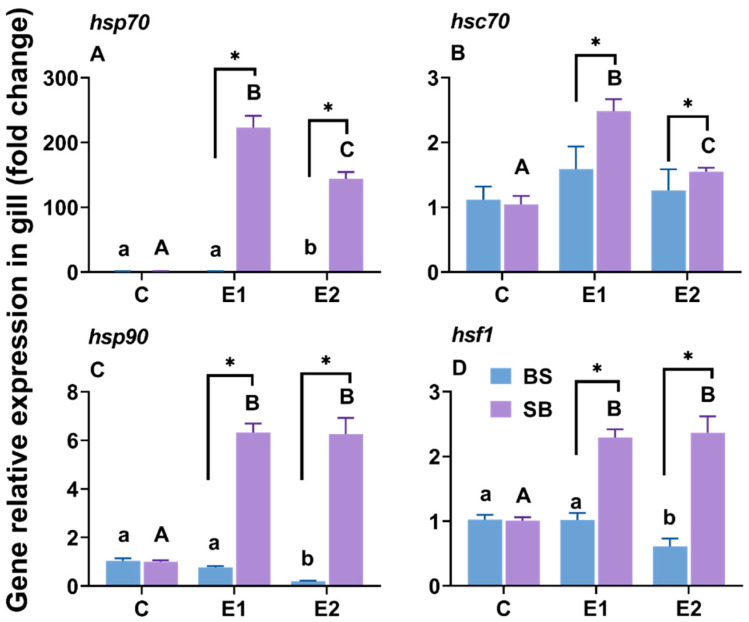
Relative mRNA expression of (**A**) *hsp70*, (**B**) *hsc70*, (**C**) *hsp90*, and (**D**) *hsf1* in gill of BS and SB hybrid sturgeon under slow heat stress. Mean values (±SEM) are shown for every group (n = 8). C: control; E1: LOE group; E2: active group. Significant differences within the BS hybrid sturgeon are denoted by different lowercase letters, while uppercase letters represent significant differences within the SB hybrid sturgeon, *p* < 0.05. Statistically significant differences (*p* < 0.05) between different hybrid sturgeon within the same group are marked by asterisks, as determined by the *t*-test.

In the gills, the reciprocal hybrids displayed opposite expression patterns compared to the liver. For SB hybrids, the expression of all measured hsp genes (*hsp70*, *hsc70*, *hsp90*) and *hsf1* was significantly elevated in the heat-stressed groups compared to the control (Figure 3). Conversely, in BS hybrids, the expression of these genes in the experimental groups was generally lower than or comparable to the control group, with *hsp70* and *hsp90* being significantly downregulated in the active group (Figure 3A,C). In both the LOE and active groups, the expression of *hsp70*, *hsp90*, and *hsf1* gene expression levels in the SB hybrid sturgeon was significantly greater than those in the BS hybrid sturgeon within the same group (consistent *p* < 0.05) (Figure 3A,C,D). Only in the LOE group of SB hybrid sturgeon was *hsc70* expression higher compared to that of BS hybrid sturgeon (Figure 3B).

### 2.3. Histological Changes in Liver Under Slow Heat Stress

**Figure 4:**
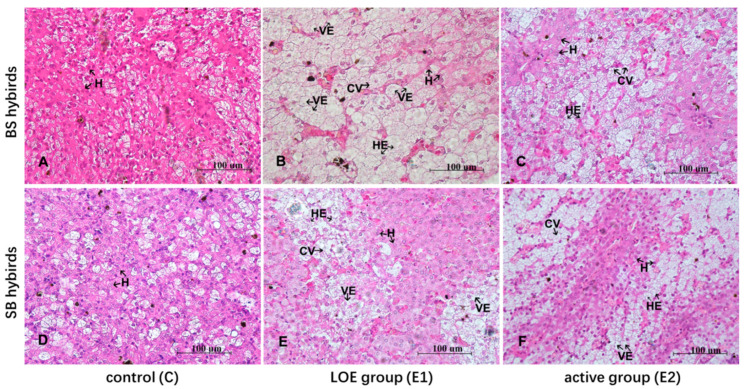
Histological changes in the hybrid sturgeon of BS and SB during in liver tissues slow heat stress under light microscopy (400×, H&E staining). (**A**): control group in BS hybrid sturgeon (C), (**B**): LOE group in BS hybrid sturgeon (E1), (**C**): active group in BS hybrid sturgeon (E2), (**D**): control group in SB hybrid sturgeon (C), (**E**): LOE group in SB hybrid sturgeon (E1), (**F**): active group in SB hybrid sturgeon (E2). CV: cellular vacuolization; H: hepatocytes; HE: hepatocellular necrose; VE, vacuole enlargement.

Hepatocytes in control group exhibited intact morphology and uniformly arranged in a tight, reticulate arrangement. Nuclei were central (Figure 4A,D). After short heat stress, hepatocyte showed the vacuole enlargement, mild nuclear displacement, hepatocellular necrosis, and asymmetric shape (Figure 4B,C,E,F). More severe hepatocyte vacuolization and the presence of anucleate hepatocytes were observed in LOE group of BS hybrid sturgeon, accompanied by cell necrosis and lysis (Figure 4B).

### 2.4. Histological Changes in Gill Under Slow Heat Stress

**Figure 5:**
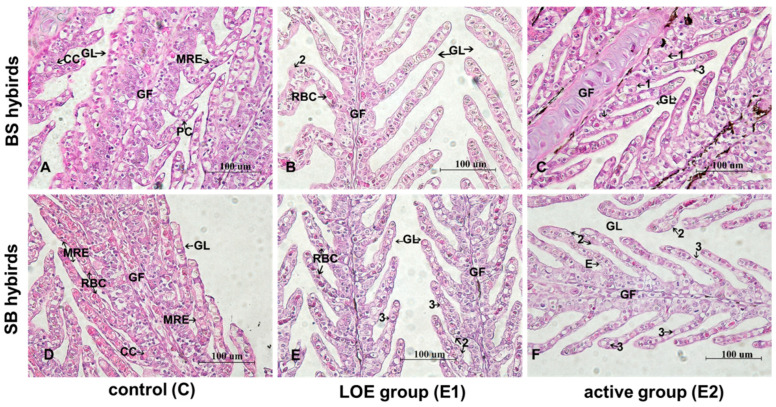
Histological changes in the hybrid sturgeon of BS and SB in gill tissues during slow heat stress under light microscopy (400×, H&E staining). (**A**): control group in BS hybrid sturgeon (C), (**B**): LOE group in BS hybrid sturgeon (E1), (**C**): active group in BS hybrid sturgeon (E2), (**D**): control group in SB hybrid sturgeon (C), (**E**): LOE group in SB hybrid sturgeon (E1), (**F**): active group in SB hybrid sturgeon (E2). CC: Chloride cell; GF: Gill filament; GL: Gill lamella; PC: pillar cell; MRE: Monolayer respiratory epithelium, RBC: Red blood cell. 1: denaturation and necrosis of stratified epithelial cells, 2: hyperplasia of MRE, 3: necrosis of MRE.

The morphology of the secondary gill lamellae exhibited normal histology (Figure 5A,D). After slow heat stress, number of vacuoles and erythrocytes presented in the gill filaments, especially in LOE group. Gill lamellae showed varying degrees of denaturation, hyperplasia, and necrosis in epithelium in experimental groups (Figure 5B,C,E,F). In the active group of BS, the thickness of gill lamellae was significantly greater than that of other experimental groups (Figure 5C).

### 2.5. Changes in Biochemical Parameters in Liver

**Figure 6:**
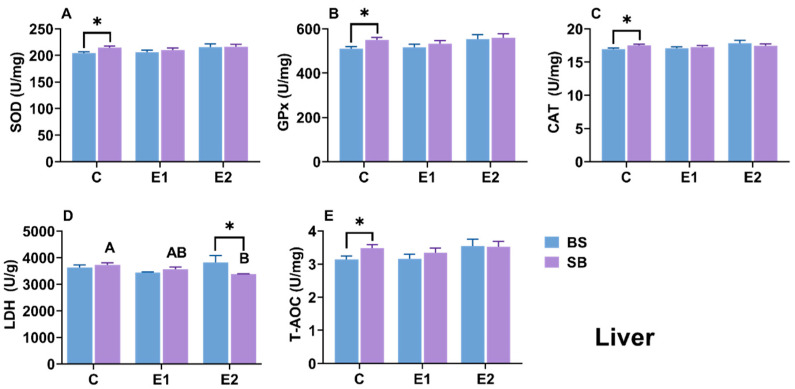
(**A**) SOD, (**B**) GPx, (**C**) CAT, (**D**) LDH activity, and (**E**) T-AOC content in the hepatic tissue of BS and SB hybrid sturgeon under slow heat stress. Mean values (±SEM) are shown for every group (n = 8). C: control; E1: LOE group; E2: active group. Uppercase letters represent significant differences within the SB hybrid sturgeon, *p* < 0.05. Statistically significant differences (*p* < 0.05) between different hybrid sturgeon within the same group are marked by asterisks, as determined by the *t*-test.

The hepatic antioxidant system (SOD, CAT, GPx) and T-AOC levels remained relatively stable, with no significant differences observed among groups for either hybrid (Figure 6A–C,E). However, metabolic enzymes showed distinct responses: LDH activity was significantly lower in the active group of SB hybrids compared to the control (*p* = 0.021), whereas it showed no significant decrease in BS hybrids (Figure 6D). In comparisons between the SB and BS within the same group, significant differences in SOD, GPx, CAT, and T-AOC were observed only in the control groups (Figure 6A–C,E). LDH differed significantly only in the active groups (*p* = 0.04) (Figure 6D).

### 2.6. Changes in Biochemical Parameters in Gill

**Figure 7:**
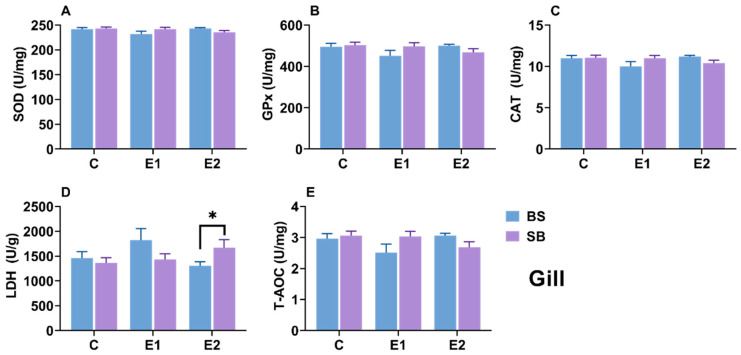
(**A**) SOD, (**B**) CAT, (**C**) LDH, (**D**) GPx activity and (**E**) T-AOC level in gill of BS and SB hybrid sturgeon under slow heat stress. Mean values (±SEM) are shown for every group (n = 8). C: control; E1: LOE group; E2: active group. Statistically significant differences (*p* < 0.05) between different hybrid sturgeon within the same group are marked by asterisks, as determined by the *t*-test.

Similar to the liver, the activities of SOD, GPx, CAT, and T-AOC in the gills showed no statistically significant differences across groups for either hybrid (Figure 7A–C,E). The only notable variation was in LDH activity, which was significantly higher in the active group of SB hybrids compared to BS hybrids (*p* = 0.05), although intra-strain comparisons did not reach statistical significance (Figure 7D). Only in the active group were significant differences in LDH enzyme activity observed between the SB and BS hybrid sturgeon (*p* = 0.05) (Figure 7D).

### 2.7. Changes in Biochemical Parameters in Kidney

**Figure 8:**
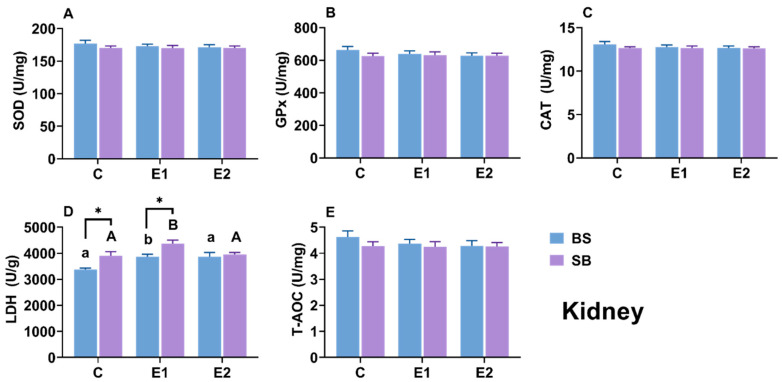
(**A**) SOD, (**B**) CAT, (**C**) LDH, (**D**) GPx activity and (**E**) T-AOC levels in kidney of BS and SB hybrid sturgeon under slow heat stress. Mean values (±SEM) are shown for every group (n = 8). C: control; E1: LOE group; E2: active group. Significant differences within the BS hybrid sturgeon are denoted by different lowercase letters, while uppercase letters represent significant differences within the SB hybrid sturgeon, *p* < 0.05. Statistically significant differences (*p* < 0.05) between different hybrid sturgeon within the same group are marked by asterisks, as determined by the *t*-test.

In the kidney, antioxidant enzymes (SOD, CAT, GPx) and T-AOC were unaffected by heat stress (Figure 8A–C,E). However, LDH activity significantly increased in the heat-stressed groups (LOE and/or active) compared to the control in both BS and SB hybrids (*p* < 0.05), suggesting a systemic metabolic adjustment (Figure 8D). When comparing between the SB and BS within the same group, significant differences in LDH enzyme activity were observed in the control and LOE groups (Figure 8D). However, no significant differences were detected in the liver or gill.

### 2.8. Changes in Cortisol, Glucose and Lactate Content in Serum

**Figure 9:**
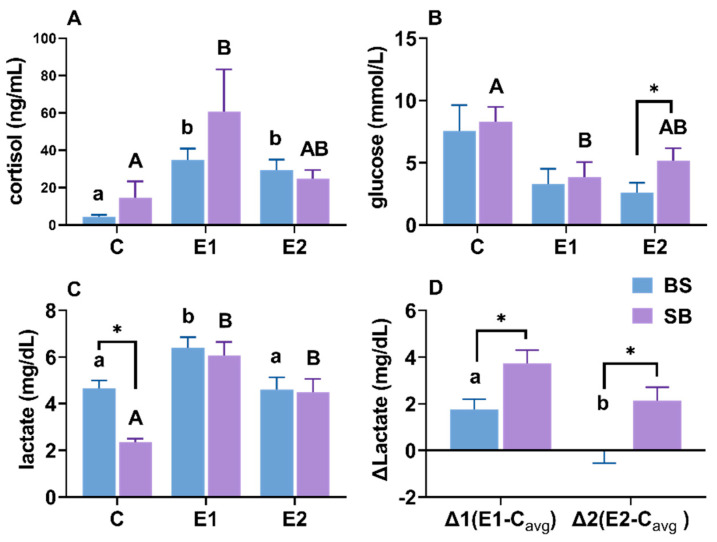
(**A**) Cortisol, (**B**) glucose, (**C**) lactate, and (**D**) δlactate (δlactate = Experimental − Control_avg_) level in serum of BS and SB hybrid sturgeon under slow heat stress. Mean values (±SEM) are shown for every group (n = 8). C: control; E1: LOE group; E2: active group. Significant differences within the BS hybrid sturgeon are denoted by different lowercase letters, while uppercase letters represent significant differences within the SB hybrid sturgeon, *p* < 0.05. Statistically significant differences (*p* < 0.05) between different hybrid sturgeon within the same group are marked by asterisks, as determined by the *t*-test.

Heat stress significantly induced a stress response, indicated by elevated serum cortisol levels in the LOE groups of both hybrids compared to controls (Figure 9A). Glucose levels generally trended downwards or remained stable, with no hyperglycemia observed (Figure 9B). Lactate metabolism differed significantly between strains: while baseline lactate varied, the net change analysis (δLactate) revealed that BS hybrids accumulated significantly less lactate than SB hybrids during stress and actively cleared it in the active group (Figure 9C,D).

### 2.9. Changes in Hepatic, Branchial, and Renal MDA Content

**Figure 10:**
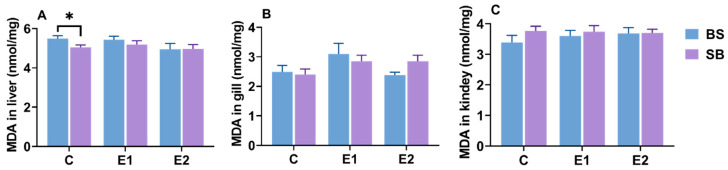
MDA level in (**A**) gill, (**B**) liver, and (**C**) kidney of BS and SB hybrid sturgeon under slow heat stress. Mean values (±SEM) are shown for every group (n = 8). C: control; E1: LOE group; E2: active group. Statistically significant differences (*p* < 0.05) between different hybrid sturgeon within the same group are marked by asterisks, as determined by the *t*-test.

No statistically significant differences in MDA levels were found in the liver, gills, and kidneys, either within the same hybrid sturgeon variety or between different varieties in the same group (Figure 10A–C).

## 3. Discussion

### 3.1. Thermal Tolerance of Reciprocal Hybrid Sturgeon

Interestingly, some sturgeon species, including juvenile and subadult white sturgeon (*A. transmontanus*) as well as Siberian sturgeon, have demonstrated the capacity to substantially increase thermal tolerance through warm acclimation, in contrast to most other fish species [33,34,35]. In this study, both BS and SB hybrid sturgeon exhibited loss of equilibrium (LOE) at temperatures above 34 °C, surpassing the LOE threshold of purebred Siberian sturgeon at 33 °C [34]. The survival curve statistics are significantly different between the hybrids (*p* < 0.05), with high survival rate in BS hybrid sturgeon (Figure 1). Furthermore, the survival rate of the active BS hybrid sturgeon group (25%) was higher than that of its parent, the Siberian sturgeon (18.3%) [34]. In preliminary trials, we attempted a similar slow heat stress test on the parent species, Amur sturgeon; however, during the heating process, more than half of the samples died, making it impossible to complete the test. Additionally, Siberian sturgeon have previously been shown to possess stronger disease resistance and higher transport survival rates compared to Amur sturgeon [36]. Taken together, these findings not only confirm the superior physiological resilience of hybrids (heterosis) but also provide clear evidence that the Siberian sturgeon maternal line confers a significant thermal tolerance advantage to its offspring. This highlights the exceptional value of Siberian sturgeon as a maternal parent for selective breeding programs aimed at developing climate-resilient sturgeon.

### 3.2. Organ-Specific Antioxidant and Metabolic Responses

Temperature fluctuations significantly impact an organism’s energy metabolism and oxygen consumption, with heat stress specifically altering oxidative balance through increased production of reactive oxygen species (ROS) [37,38]. Excessive ROS levels can lead to oxidative imbalance, resulting in damage to enzyme activity, protein structures, and lipid fluidity [39]. In this study, the primary antioxidant system (e.g., SOD, CAT, GPx, T-AOC) remained surprisingly stable across all three tissues in both hybrids. This suggests that under the condition of slow heat stress, the sturgeon did not rely on this system but instead employed an alternative metabolic strategy. This alternative strategy appeared to be the redistribution of lactate metabolism, indicated by changes in lactate dehydrogenase (LDH) activity. The most prominent shared response was a significant elevation of LDH activity in the kidneys of both BS and SB stressed groups (Figure 8D). This systemic renal response indicates a shift toward anaerobic metabolism and highlights the kidney’s compensatory role in processing lactate accumulation. This reliance on metabolic adaptation rather than antioxidant activation is consistent with responses to gradual, slow stress [40] and has been observed in Chinese sturgeon (*A. sinensis*) [41], olive flounder (*Paralichthys olivaceus*) [42], shortnose sturgeon [26], and Siberian sturgeon [34]. Crucially, however, the two hybrids showed completely divergent strategies in their other primary organs (Figure 6D and Figure 7D).

It is worth noting that significant baseline differences were observed between the reciprocal hybrids prior to heat stress. In the hepatic tissue of the control groups, the SB hybrid exhibited significantly higher constitutive levels of SOD, GPx, CAT, and T-AOC compared to the BS hybrid (Figure 6). While high antioxidant levels are typically associated with robust defense, in this context, the higher baseline in SB hybrids may indicate a higher physiological cost of maintenance or a state of elevated basal oxidative stress even under optimal conditions. In contrast, the BS hybrid (inheriting the *A. baerii* maternal lineage) maintained lower basal enzyme activities. This lower “cost of living” under control conditions suggests a more efficient basal metabolism, potentially providing the BS hybrid with greater physiological plasticity and energy reserves to mobilize specific coping mechanisms (such as the lactate clearance pathway discussed below) when challenged by thermal stress. This divergence in baseline status is likely another manifestation of the maternal effect, influencing the overall thermal resilience of the offspring.

When comparing the hybrids directly: the BS hybrid exhibited significantly higher LDH activity in the Liver while the SB hybrid exhibited significantly higher LDH activity in the Gills. These contrasting patterns strongly suggest that the BS hybrid relies mainly on hepatic metabolic adjustment. In contrast, the SB hybrid depends on peripheral compensation in the gills. Overall, these LDH dynamics highlight that metabolic redistribution is the dominant strategy for sturgeon homeostasis under slow heat. This strategic difference aligns perfectly with our other findings: the BS hybrid’s liver-centric strategy (mirroring its hepatic HSP response, discussed below) was survivable, whereas the SB hybrid’s gill-centric compensation (mirroring its catastrophic gill damage) led to fatal respiratory and osmotic failure. This conclusion is strongly corroborated by the serum lactate analysis (Figure 9D). While baseline lactate levels differed, the analysis of δlactate (net change) reveals the true metabolic capacity of the hybrids. During stress (E1), the net increase in lactate was significantly smaller in BS hybrids, and in the active group (E2), BS hybrids were actively clearing lactate (a negative δ) while SB hybrids were still accumulating it. This provides definitive evidence that the BS hybrid’s ‘hepatic adjustment’ strategy—relying on the liver, the primary organ for gluconeogenesis—was highly effective at processing and removing lactate from the system. Conversely, the SB hybrid’s ‘peripheral compensation’ in the gills was an ineffective stress response, leading to uncontrolled systemic lactate accumulation, metabolic acidosis, and ultimately, mortality. This superior metabolic efficiency is a key component of the enhanced thermal tolerance conferred by the Siberian sturgeon maternal line.

Interestingly, a divergence was observed between the significant histological damage (Figure 4 and Figure 5) and the stable MDA levels (Figure 10). While MDA is a classic marker for lipid peroxidation, its stability suggests that lipid peroxidation was not the primary driver of cell death under the slow heat stress regime. Instead, the observed tissue necrosis and vacuolization were likely driven by proteotoxicity and metabolic disruption. First, the significant upregulation of hsp genes (Figure 2 and Figure 3) indicates that the cellular machinery was heavily engaged in combating protein denaturation and aggregation. When this chaperone capacity is exceeded, protein collapse leads to structural failure and necrosis, independent of lipid oxidation [43]. Second, the systemic increase in LDH and lactate accumulation (Figure 8 and Figure 9) points to metabolic acidosis and energy depletion. According to the principle of oxygen- and capacity-limitation of thermal tolerance (OCLTT), the transition to anaerobic metabolism (indicated by lactate buildup) marks the physiological limit of thermal tolerance, leading to systemic failure and cell death before substantial oxidative damage accumulates [44]. Therefore, in this slow heating model, the sturgeon likely maintained oxidative homeostasis (stable MDA) but succumbed to the cumulative structural and metabolic costs of thermal adaptation.

### 3.3. Heat Shock Protein Gene Expression

HSP70 and HSP90 are crucial for mitigating heat stress-induced oxidative damage [45]. These functions have been observed in various species, including fathead minnow (*Pimephales promelas*) [46], larval green sturgeon (*A. medirostris*) [47], juvenile shortnose sturgeon [26], Amur sturgeon [48], Russian sturgeon [4], and Yangtze sturgeon (*A. dabryanus*) [49]. In this study, a gradual increase in water temperature significantly upregulated *hsp* gene family expression in the hepatic tissue of BS hybrid sturgeon and the gills of SB hybrid sturgeon. However, in the gills of BS hybrid sturgeon and the liver of SB hybrid sturgeon, the expression of *hsp* family genes in the experimental groups was either similar to or reduced compared to the expression profile in the control group (Figure 2A–C and Figure 3A–C). Similar patterns have been recorded in redband trout (*Oncorhynchus mykiss*) [50], lake sturgeon (*A. fulvescens*) [27], and Siberian sturgeon [36]. Notably, *hsp70* expression was higher in the liver than gills (Figure 2A and Figure 3A), consistent with observations in green sturgeon [51]. However, *hsp90* expression in kaluga (*H. dauricus*) under heat stress either matched or exceeded *hsp70* levels [36,52]. Acute heat shock has been shown to significantly upregulate *hsc70* expression in numerous species, including zebrafish [53], sea bream [28], turbot (*Scophthalmus maximus*) [54], and pikeperch (*Sander lucioperca*) [55]. In the current study, *hsc70* expression was elevated in the liver of BS hybrid sturgeon from the LOE group and in the branchial tissues of SB hybrid sturgeon (Figure 3B,C). The expression pattern of *hsc70* in BS hybrid sturgeon is the same as that in Siberian sturgeon [34].

Species such as zebrafish [56], orange-spotted grouper (*Epinephelus coioides*) [57], and Russian sturgeon [4] exhibited tissue-specific differences in HSF1 regulation in response to elevated temperatures. Additionally, *hsf1* expression varied across developmental stages and thermal conditions [58]. In this study, stress-induced HSP expression in both liver and gills was regulated by *hsf1* (Figure 2D and Figure 3D), implying potential roles for *hsf1* beyond classical HSP regulation, including HSP70-independent anti-inflammatory responses [59]. However, *hsf1* exhibited differential expression exclusively in the gills of SB hybrid sturgeon, indicating that other *hsp* isoforms may involve HSP regulation in different tissues. Further research is required to explore the molecular foundations of these tissue-specific stress responses and their associated tolerance mechanisms.

When comparing the two hybrids, we found clear tissue-specific differences: in the BS hybrids, *hsp genes* were mainly up in the liver (Figure 2), while in the SB hybrids, they were up in the gills (Figure 3). This shows that there are organ-specific responses to slow-heating stress in hybrids. From the histological observation, the BS hybrids showed more vacuolated and anucleated liver cells—suggesting their liver function was under heavier strain compared to the SB hybrid (Figure 4B,E) while gill lamella of BS hybrid appear to have suffered less damage than SB hybrids’ (Figure 5C,F). This presents an apparent paradox: the BS hybrid survived, yet its liver was more damaged. This, however, likely reflects its inherited survival strategy. The SB hybrid, with its critical respiratory organ (gills) compromised first, suffered rapid systemic failure and mortality. In contrast, the BS hybrid (inheriting from the Siberian sturgeon maternal line) appears to mobilize the liver as the primary stress-response organ-a strategy consistent with the high hepatic stress response observed in the parent Siberian sturgeon [34]. While this results in temporary hepatic damage, it secures the function of critical organs like the gills, allowing for higher overall survival. This highlights the superior resilience of the strategy inherited from the Siberian sturgeon maternal line.

These distinct expression patterns suggest that Hsps may fulfill divergent physiological roles depending on the tissue context. In the hepatic tissue of the heat-tolerant BS hybrid, the significant upregulation of *hsp* family genes likely functioned primarily as active molecular chaperones. By enhancing protein stability and facilitating the repair of denatured proteins, this robust hepatic response enabled the maintenance of critical metabolic functions (e.g., lactate clearance) despite the thermal insult.

Conversely, the downregulated or stable *hsp* gene expression in the gills of BS hybrids may indicate a reduced perception of stress or, more likely, a lower degree of proteotoxic damage in this specific tissue. This aligns with the histological findings where BS gills remained relatively intact compared to SB gills. In contrast, the high *hsp gene* expression in the gills of the heat-sensitive SB hybrid likely served as a marker of severe local tissue stress and structural failure rather than an effective adaptation. Thus, the ability to mobilize HSPs for hepatic protection while sparing the respiratory organs appears to be a key mechanism underlying the superior thermal tolerance of the BS hybrid.

### 3.4. Hormonal and Energy Metabolism Changes

In the present study, heat stress significantly elevated cortisol levels, ranging from 9.97 to 61.43 ng/mL in BS hybrid sturgeon and 12.91 to 183.23 ng/mL in SB hybrid sturgeon (Figure 9B). These levels exceed those documented in earlier studies, with 107.89 ± 88.0 ng/mL reported for Adriatic sturgeon (*A. naccarii*) at 25 °C [60] and 50 ng/mL detected in Russian sturgeon (*A. gueldenstaedtii*) [61], confirming the severity of the stress response. Interestingly, while cortisol typically promotes gluconeogenesis [62], we did not observe the expected rise in plasma glucose. Instead, plasma glucose levels decreased, particularly in BS hybrids, suggesting a rapid depletion of energy reserves that outpaced gluconeogenic capacity (Figure 9A,B). Resting plasma glucose levels in pallid sturgeon (*Scaphirhynchus albus*) were previously reported to be lower (46.1–48.4 mg/dL) [63] than control group of the present study, which ranged from 7.56 mmol/L (136.08 mg/dL) to 8.28 mmol/L (149.04 mg/dL) (Figure 9B).

Under heat stress, plasma glucose levels decrease, particularly in BS hybrid sturgeon. Similar decreases were reported in Adriatic sturgeon when temperature raised from 17 °C to 25 °C [60], shortnose sturgeon during 10 °C to 20 °C shifts [64], and Siberian sturgeon after water temperature increased from 21 °C to 34 °C [34]. Conversely, other studies have documented increased glucose levels under more rapid temperature changes. For instance, glucose levels raised when water temperature increased by 8 °C/h [31], 9 °C/h [65], 12 °C/h, and 15 °C/h [26]. However, glucose levels decreased under slower temperature changes, such as 6 °C/h [26,64], 1 °C over 8 h in Siberian sturgeon [34] and in the present study. These results indicate that the magnitude and rate of temperature changes are crucial in influencing sturgeon metabolism when exposed to heat stress.

### 3.5. Implications for Aquaculture and Future Research

Taken together, these findings suggest that reciprocal hybrid sturgeons employ distinct mechanisms to cope with slow thermal stress. The BS hybrid adopted a ‘liver-centric’ response, whereas the SB hybrid relied on a ‘gill-centric’ response. Although this resulted in more severe liver damage in BS hybrids, the gill damage in SB hybrids was catastrophic. Given the critical respiratory function of gills, this severe gill stress likely led to irreversible damage and rapid mortality. This observation explains why BS hybrid sturgeon, by protecting their critical respiratory function at the expense of the liver, ultimately survived longer than the SB hybrids.

Studies have demonstrated that dietary supplementation with selenoproteins could reduce MDA levels in the liver, enhance GPx activity, and promote liver repair [66]. Similarly, dietary vitamin E has been found to significantly increase the antioxidant enzyme activity profiles, including GPx, SOD, CAT, nitroblue tetrazolium (NBT) reductase, and lysozyme, while also enhancing phagocytic activity and reducing MDA levels [67]. Phytobiotics, derived from plants or their extracts, have emerged as promising aquafeed supplements to improve growth, enhance immune responses, potentiate antioxidant status, and increase disease resistance in fish. For instance, plant extracts such as curcumin and quercetin have been widely investigated for their anti-inflammatory properties, particularly in mitigating inflammation in gill and liver tissues [68]. Curcumin has been shown to alleviate gill inflammation in cyprinid *Puntius sophore* by inhibiting the nuclear factor kappa B (NF-κB) signaling pathway, thereby improving gill tissue functionality [69]. Furthermore, silymarin has been proven effective in reducing liver damage by enhancing mitochondrial function in hepatocytes [70]. These findings provide valuable insights that could be further explored in sturgeon research, particularly in aquatic applications in aquaculture. Incorporating such dietary supplements into sturgeon feeding regimes could contribute to improving health status, enhancing resilience to environmental stressors, and promoting sustainable industry development of aquaculture.

## 4. Materials and Methods

### 4.1. Experimental Fish

All sixty-six-month-old hybrid sturgeons (*A. baerii* ♀ × *A. schrenckii* ♂, BS hybrid; *A. schrenckii* ♀ × *A. baerii* ♂, SB hybrid) were obtained from the Sturgeon Seed Farm of the State (Beijing, China). Prior to the experiment, all fish were acclimated at 21 °C for 7 days under standard conditions, with feedings twice daily provided from Beijing HANYE Co., Ltd., China. After acclimation, sixty individuals of each hybrid sturgeon, with comparable weights (BS: 474.3 ± 40.2 g; SB, 471.0 ± 42.1 g), were randomly allocated to three tanks (about 0.9 m in inner diameter, 0.7 m in water depth, and a total volume of 0.45 m^3^), with 20 fish per tank. Water exchange was performed using temperature-equilibrated to maintain stability. During the entire trial (from 21 °C to LOE), vigorous aeration was provided to maintain dissolved oxygen levels > 6 mg/L, mitigating the effect of hypoxia often associated with high temperatures. Water quality parameters (ammonia and nitrite) were monitored daily to ensure they remained within safe limits. The measurements were monitored using an HQd Portable Meter (HACH Co., Loveland, CO, USA).

### 4.2. Slow Heat Stress Exposure and Sampling

To establish baseline conditions, fish were fasted for 24 h prior to heat stress. Eight fish were randomly sampled as controls from the three tanks before the temperature was raised. After control sampling, the water temperature of each stress group increased by 1 °C per 8 h until individuals exhibited loss of equilibrium (LOE), defined as the inability to maintain a dorsal-up orientation position for a continuous duration of 30 s. To achieve precise thermal control, the water temperature was regulated using intelligent thermostats installed in each tank. The temperature was programmed to increase by 1 °C over a 1 h period, followed by a 7 h maintenance phase, resulting in a net increase of 1 °C every 8 h. Sampling was conducted based on physiological endpoints. The LOE group (E1, n = 8) consisted of the first eight individuals to exhibit LOE; each was sampled immediately individually upon losing equilibrium to capture the acute stress state. The Active group (E2, n = 8) was sampled from the remaining swimming individuals at the same time point (when the 8th LOE fish was sampled) to serve as a biologically active comparison under identical thermal conditions. The water temperature was recorded immediately upon the fish reaching LOE. All animal handling and experimental procedures complied with the Chinese Council on Animal Care guidelines. During sampling, the fish were anesthetized with 200 ppm MS222. Blood was obtained from each fish via tail vein puncture, centrifuged at 4 °C and 1500× *g* for 10 min, and serum stored at −80 °C for subsequent analysis. A portion of the hepatic tissue and the second gill arch were fixed in 4% paraformaldehyde for histological analysis. The kidney, residual hepatic tissue, and gill tissues were flash-frozen in liquid nitrogen and subsequently preserved at −80 °C for further assays.

### 4.3. RNA Extraction and Reverse Transcription

Total RNA was extracted from liver and gill tissues using the RNeasy Plus Mini Kit (Cat No. 74134, Qiagen, Germany). During the extraction process, genomic DNA was removed using the RNase-Free DNase Set (Cat No. 79254, Qiagen, Germany) according to the manufacturer’s protocol. RNA concentration and purity were measured using a Nanodrop 2000 spectrophotometer (Thermo, Waltham, MA, USA), and only high-quality RNA with an A260/A280 ratio between 1.8 and 2.1 was selected for subsequent reverse transcription. For each sample, reverse transcription of 4 μg of RNA was performed using an oligo (dT) primer and the SuperScript III System (Cat No. 18080051, Invitrogen, Carlsbad, CA, USA).

### 4.4. Real-Time Quantitative PCR

All primers applied in real-time quantitative PCR (qPCR) were designed using Primer 3 (version 0.4.0, available at https://bioinfo.ut.ee/primer3-0.4.0/ (accessed on 1 November 2025)) and listed in Table 1. The specificity of the primer sets was validated through the molecular cloning and sanger sequencing of the obtained single amplicons. QPCR was performed using the dual internal reference method and candidate reference genes including *bactin*, *ef1a*, *rpl13*, *rpl6*, *18s*, *gapdh* were evaluated in preliminary experiments [71]. The expression levels of target genes were finally normalized to geometric mean of the reference genes *ef1a* and *bactin* [72] based on preliminary experiment. Relative gene expression was quantified using the 2^-ΔΔCt^ method [73].

QPCR amplification was performed using QuantStudio 3 system (Thermo, Waltham, MA, USA) with the following thermal cycling conditions: initial incubation at 50 °C for 2 min, enzyme activation at 95 °C for 5 min, followed by 40 cycles of denaturation at 95 °C for 30 s, annealing at 60 °C for 30 s, and extension at 72 °C for 30 s. Melting curve analysis was conducted at the end of the amplification to verify reaction specificity. Negative controls were included by replacing the cDNA template with sterile deionized water to ensure the absence of contamination. The final reaction volume for qPCR was 20 μL, consisting of 1 μL of 10-fold diluted cDNA, 10 μL of 2× SYBR Green qPCR master mix (A25742, ABI, Carlsbad, CA, USA), 0.2 μL of each primer (10 μM), and 8.6 μL of nuclease-free water. All samples were analyzed in technical triplicates to ensure reproducibility.

### 4.5. Histological Procedure

Fixed liver and gill tissues were dehydrated through a graded ethanol series, embedded in paraffin, and sectioned at a thickness of 7 μm. Sections were stained with hematoxylin and eosin using standard histological procedures with the Leica pathological section system. Tissue morphology was examined under an optical microscope, and images were captured using an Olympus BX51 microscope (Olympus, Hachiōji, Japan).

### 4.6. Liver, Gill, Kidney, and Serum Biochemical Analyses

Tissue homogenates from liver, gill, and kidney were prepared by diluting samples 1:9 (*w*/*v*) with ice-cold NaCl (0.68%). After centrifugation at 3000× *g* for 10 min at 4 °C, the supernatant was collected for biochemical analyses. Serum samples were pooled for direct analysis. All assays, except serum cortisol, used reagent kits from Nanjing Jiancheng Bioengineering Institute, Nanjing, China. (Item: T-AOC: A015-1; SOD: A001-1; CAT: A007-1-1; LDH: A019-2; GPx: S0056; MDA: A003-1; Glucose: A154-1-1; lactate: A019-2-1). Serum cortisol was determined using a competitive radioimmunoassay with iodine-125 ([^125^I]). In this assay, cortisol from the standards or samples competes with radiolabeled ^125^I-cortisol for limited antibody binding sites. The bound ^125^I-cortisol is inversely proportional to cortisol concentration. A standard curve was generated by plotting the binding rates against known cortisol concentrations, allowing the cortisol levels in the test samples to be calculated.

### 4.7. Data Analysis

Statistical analyses were performed using IBM SPSS (version 26.0). Kaplan–Meier survival curves were plotted, and log-rank tests were conducted to compare the survival outcomes between two hybrid sturgeon groups. The analysis was conducted on a cohort of 44 individuals per group, calculated by excluding the control (n = 8) and active sampling (n = 8) groups from the initial population (n = 60). Individuals that exhibited Loss of Equilibrium (LOE) or mortality were recorded as events. The individuals that remained alive at the end of the 24 h post-stress observation period were included in the analysis as right-censored data.

Prior to analysis, data normality and homogeneity of variance were verified. Comparisons among the three groups (Control, LOE, and Active) within the same hybrid strain were conducted using one-way ANOVA followed by Tukey’s post hoc test for normally distributed data. For data that did not meet parametric assumptions, the non-parametric Kruskal–Wallis test was employed, followed by the Mann–Whitney U test for pairwise comparisons. To compare differences between the two hybrid strains (BS vs. SB) within the same treatment group (Control, LOE, or Active), an independent samples *t*-test was used for normally distributed data, while the Mann–Whitney U test was used for non-normally distributed data Results are presented as mean ± SEM, with statistical significance established at *p* < 0.05.

## 5. Conclusions

This study confirms that the BS hybrid (*A. baerii* ♀ × *A. schrenckii* ♂) exhibits superior thermal tolerance and survival compared to its reciprocal SB cross. This advantage is rooted in its unique, liver-centric tissue-specific stress response (e.g., hepatic *hsp* upregulation), which contrasts sharply with the fatal gill-centric failure observed in the SB hybrid. This finding strongly underscores the critical impact of maternal lineage on the heat resilience of hybrids. When combined with our previous research establishing the high intrinsic thermal tolerance and robust hepatic antioxidant mechanisms of the Siberian sturgeon parent itself, this study provides new evidence that this superior thermal resilience is effectively transmitted via the maternal line. Therefore, the Siberian sturgeon maternal line (as represented in ‘Jinglong No. 1’) is not only a robust choice for current aquaculture but also a prime genetic resource for future selective breeding programs aimed at developing climate-resilient sturgeon.

## Figures and Tables

**Table 1:** Primer sets used for quantitative PCR.

Gene Name	Gene Symbol	*Primers (5′-3′)*	Length (bp)	R^2^	Efficiency %	*Accession Number*
*b-actin*	*bact*	*F: TGCCATCCAGGCTGTGCT*	215	0.997	93.827	*JX027376.1*
		*R: TCTCGGCTGTGGTGGTGAAG*				
*elongation factor 1 alpha*	*ef1a*	*F: GGACTCCACTGAGCCACCT*	89	0.989	108.07	*JQ995144.1*
		*R: GGGTTGTAGCCGATCTTCTTG*				
*heat shock transcription factors 1*	*hsf1*	*F: ATTAGCCACGGTCACACACA*	158	0.989	96.913	*XM_033993892.2*
		*R: GAAACAGCAGTCACCTTCCC*				
*heat shock cognate 70*	*hsc70*	*F: CCTCTAAGAGAGCGGCTGAG*	100	0.993	93.716	*XM_033995557.2*
		*R: TTACTGCAGCTCGGAAGAGAG*				
*heat shock protein 70*	*hsp70*	*F: CCCTACCATCGAGGAGGTTG*	168	0.995	91.279	*HM348777.1*
		*R: AATGACCAGCGTTGGCTTAC*				
*heat shock protein 90*	*hsp90*	*F: TGAGGATGTTGGCTCTGATG*	110	0.999	96.250	*JX477807.1*
		*R: AGATGGGCTTGGTCTTGTTC*				

## Data Availability

The original contributions presented in this study are included in the article. Further inquiries can be directed to the corresponding authors.
